# 4-(9-Anthr­yl)-1-(4-methoxy­phen­yl)spiro­[azetidin-3,9′-xanthen]-2-one

**DOI:** 10.1107/S1600536808010908

**Published:** 2008-04-26

**Authors:** Mehmet Akkurt, Selvi Karaca, Aliasghar Jarrahpour, Edris Ebrahimi, Orhan Büyükgüngör

**Affiliations:** a Department of Physics, Faculty of Arts and Sciences, Erciyes University, 38039 Kayseri, Turkey; b Department of Chemistry, College of Sciences, Shiraz University, 71454 Shiraz, Iran; c Department of Physics, Faculty of Arts and Sciences, Ondokuz Mayıs University, 55139 Samsun, Turkey

## Abstract

In the title mol­ecule, C_36_H_25_NO_3_, the β-lactam ring is essentially planar, with a dihedral angle of 3.3 (2)° between the two separate three-atom N/C/C planes. The β-lactam ring makes dihedral angles of 28.45 (14), 87.4 (1) and 51.8 (1)° with the mean planes of the benzene, xanthene and anthracene ring systems, respectively. In addition to a weak intra­molecular C—H⋯N hydrogen bond, the crystal structure is stabilized by two weak inter­molecular C—H⋯O hydrogen bonds.

## Related literature

For related literature, see: Alonso *et al.* (2001 ▶, 2002 ▶); Bycroft *et al.* (1988 ▶); Fukuda & Endo (1988 ▶); Jarrahpour & Khalili (2007 ▶); Kambara & Tomioka (1999 ▶); Pinder & Weinreb (2003 ▶); Sheehan *et al.* (1978 ▶); Skiles & McNeil (1990 ▶); Akkurt *et al.* (2006 ▶, 2007 ▶); Nardelli (1995 ▶); Pınar *et al*. (2006 ▶); Allen *et al.* (1987 ▶); Cremer & Pople (1975 ▶); Spek (2003 ▶).
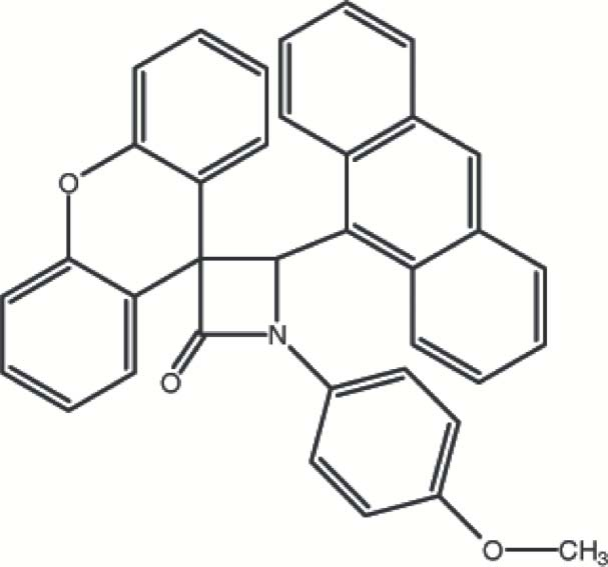


## Experimental

### 

#### Crystal data

C_36_H_25_NO_3_
*M* *_r_* = 519.57
Monoclinic, 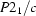

*a* = 13.7629 (9) Å
*b* = 10.5845 (4) Å
*c* = 20.5777 (12) Å
β = 114.768 (5)°
*V* = 2721.9 (3) Å^3^
*Z* = 4
Mo *K*α radiation
μ = 0.08 mm^−1^
*T* = 293 (2) K
0.61 × 0.33 × 0.16 mm

#### Data collection

Stoe IPDS-2 diffractometer
Absorption correction: none
19756 measured reflections
5559 independent reflections
3359 reflections with *I* > 2σ(*I*)
*R*_int_ = 0.100

#### Refinement

*R*[*F* ^2^ > 2σ(*F* ^2^)] = 0.061
*wR*(*F* ^2^) = 0.133
*S* = 1.03
5559 reflections
361 parameters
H-atom parameters constrained
Δρ_max_ = 0.13 e Å^−3^
Δρ_min_ = −0.18 e Å^−3^

### 

Data collection: *X-AREA* (Stoe & Cie, 2002 ▶); cell refinement: *X-AREA*; data reduction: *X-RED32* (Stoe & Cie, 2002 ▶); program(s) used to solve structure: *SIR97* (Altomare *et al*., 1999 ▶); program(s) used to refine structure: *SHELXL97* (Sheldrick, 2008 ▶); molecular graphics: *ORTEP-3 for Windows* (Farrugia, 1997 ▶); software used to prepare material for publication: *WinGX* (Farrugia, 1999 ▶).

## Supplementary Material

Crystal structure: contains datablocks global, I. DOI: 10.1107/S1600536808010908/lh2616sup1.cif

Structure factors: contains datablocks I. DOI: 10.1107/S1600536808010908/lh2616Isup2.hkl

Additional supplementary materials:  crystallographic information; 3D view; checkCIF report

## Figures and Tables

**Table 1:** Hydrogen-bond geometry (Å, °)

*D*—H⋯*A*	*D*—H	H⋯*A*	*D*⋯*A*	*D*—H⋯*A*
C8—H8⋯O3^i^	0.93	2.41	3.317 (4)	164
C18—H18⋯O1^ii^	0.93	2.58	3.419 (3)	150
C25—H25⋯N1	0.93	2.28	2.916 (3)	125

Symmetry codes: (i) 
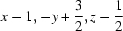
; (ii) 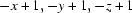.

**Fig. 1.**
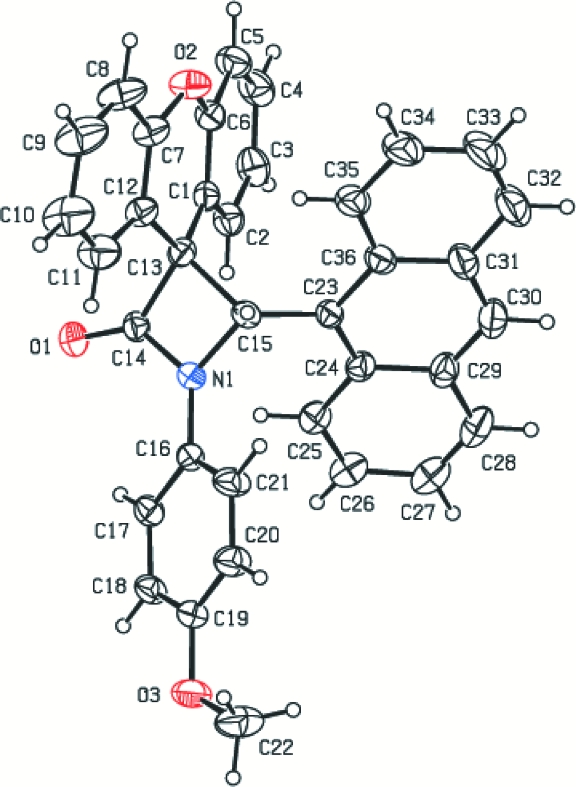
The title molecular structure, with the atom-numbering scheme and 30% probability displacement ellipsoids

**Fig. 2.**
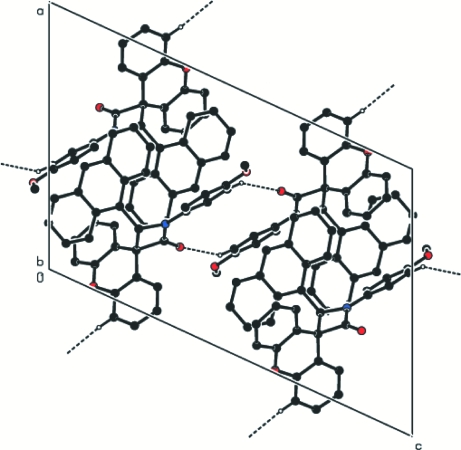
A view along the b axis of the packing and hydrogen bonding interactions as dashed lines. H atoms not involved in hydrogen bonding have been omitted.

## supplementary crystallographic information

### Comment

Although various examples of β-lactams with spiro structures derived from penicillins or cephalosporins have been reported (Bycroft *et al.*, 1988), only a few examples of simple spiro β-lactams are known today (Fukuda & Endo, 1988; Jarrahpour & Khalili, 2007). Spiro cyclic β-lactams behave as β-turn mimetics (Alonso, Lopez-Ortiz *et al.*, 2001), and as cholesterol absorption inhibitors (Kambara & Tomioka, 1999). They can act as antiviral (Skiles & McNeil, 1990), antibacterial agents (Sheehan *et al.*, 1978), and they are precursors of α,α-disubstituted β-amino acids (Alonso *et al.*,  2002). The spiranic β-lactam moiety is present in chartellines, a family of marine natural products (Pinder & Weinreb, 2003).

In the title molecule (Fig. 1), the bond lengths are within the normal ranges (Allen *et al.*,  1987). The angles exocyclic to and those involving the β-lactam moiety (C13–C15/N1) are comparable with the values in our previously reported structures containing the β-lactam group (Pınar *et al.*, 2006; Akkurt *et al.*, 2007; Akkurt *et al.*, 2006).

The β-lactam ring is nearly planar, with a maximum deviation of 0.017 (2) Å from the ring.  The dihedral angle between the N1/C1/C2 and N1/C2/C3 planes is 176.7 (2)° (Nardelli, 1995). The planarity is mainly due to the *sp*^2^ hybridization of atoms C14 and N1. Atom O1 lies essentially in the β-lactam ring plane, with a deviation of -0.005 (2) Å. The dihedral angle between the benzene ring C16—C21 attached at N1 and the β-lactam ring is 28.45 (14)°.

In the xanthene ring system, attached at C13, the benzene rings (C1–C6) and (C7–C12) are almost co-planar, forming a dihedral angle of 12.4 (1)° with each other. The central ring, C1/C6/O2/C7/C12/C13, is not planar, with puckering parameters: Q_T_ = 0.195 (2) Å, θ = 100.8 (6)° and φ = 7.2 (8)° (Cremer & Pople, 1975). The mean planes of the xanthene ring system forms the dihedral angles of 87.4 (1)°, and 59.2 (1)°, with the β-lactam ring and the benzene ring C16–C21, respectively.

The anthracene ring system, attached at C15, is almost planar, with maximum deviations of 0.095 (2) Å for C23, -0.061 (2) Å for C25, -0.052 (2) Å for C26 and 0.041 (3) Å for C28. It forms dihedral angles of 51.8 (1)°, 74.2 (1)° and 62.91 (7)°, with the β-lactam ring, benzene ring (C16–C21) and the mean plane of the xanthene ring system, respectively.

The crystal structure of the title compound is stabilized by two intermolecular C—H···O and an intramolecular C—H···N hydrogen bonding interactions (Table 1 and Fig. 2).

### Experimental

A mixture of the Schiff base (*E*)—*N*-(anthracen-9-ylmethylene)-4-methoxyaniline (0.3 g, 1.45 mmol) and triethylamine (0.73 g, 7.27 mmol), 9*H*-xanthen-9-carboxylic acid (0. 49 g, 2.18 mmol) and tosyl chloride (0.42 g, 2.18 mmol) in CH_2_Cl_2_ (15 ml) was strirred at room temperature for 24 h. Then it was washed with HCl 1 N (20 ml) and saturated sodium bicarbonate solution (20 ml), brine (20 ml), dried (Na_2_SO_4_) and the solvent was evaporated to give the crude product as light yellow crystals which was then purified by recrystalization from ethyl acetate (yield 25%) [m.p. 470–472 K]. IR (CHCl_3_, cm^-1^): 1747.0 (CO β-lactam). ^1^H-NMR d (p.p.m.): 3.64 7(s, 3H, OCH_3_), 6.298 (s, 1H, 4), 6.511–8.826(m, ArH, 21H). ^13^C-NMR d (p.p.m.): 64.35 (OCH_3_), 62.84 (C-3), 75.68 (C-4), 116–152 (aromatic carbon), 167.34 (CO β-lactam). Analysis calculated for C_36_H_25_NO_3_: C 83.22, H 4.85, N 2.70%. Found: C 83.95, H 4.90, N 2.82%.

### Refinement

H atoms were included in ideal positions and refined by using a riding model, with C–H = 0.93 Å for aromatic, 0.96 Å for methyl and 0.98 Å for methine, and with *U*_iso_ = 1.2 or 1.5*U*_eq_(C).

During the refinement of the structure, electron density peaks were located that were believed to be disordered solvent molecules (water from reactio solvent). The SQUEEZE option in PLATON (Spek, 2003) indicated there was a solvent cavity of volume 34 Å^3^ containing approximately 1.5 electrons. In the final cycles of refinement, this contribution to the electron density was removed from the observed data. The density, the F(000) value, the molecular weight and the formula are given without taking into account the results obtained with the SQUEEZE option PLATON (Spek, 2003).

### Crystal data

**Table:**

C_36_H_25_NO_3_	*F*_000_ = 1088
*M**_r_* = 519.57	*D*_x_ = 1.268 Mg m^−^^3^
Monoclinic, *P*2_1_/*c*	Mo *K*α radiation λ = 0.71073 Å
Hall symbol: -P 2ybc	Cell parameters from 23499 reflections
*a* = 13.7629 (9) Å	θ = 1.5–28.1º
*b* = 10.5845 (4) Å	µ = 0.08 mm^−^^1^
*c* = 20.5777 (12) Å	*T* = 293 (2) K
β = 114.768 (5)º	Prism, light yellow
*V* = 2721.9 (3) Å^3^	0.61 × 0.33 × 0.16 mm
*Z* = 4	

### Data collection

**Table:**

Stoe IPDS-2 diffractometer	3359 reflections with *I* > 2σ(*I*)
Monochromator: plane graphite	*R*_int_ = 0.100
Detector resolution: 6.67 pixels mm^-1^	θ_max_ = 26.5º
*T* = 293(2) K	θ_min_ = 1.6º
ω scans	*h* = −17→17
Absorption correction: none	*k* = −13→13
19756 measured reflections	*l* = −25→25
5559 independent reflections	

### Refinement

**Table:**

Refinement on *F*^2^	Secondary atom site location: difference Fourier map
Least-squares matrix: full	Hydrogen site location: inferred from neighbouring sites
*R*[*F*^2^ > 2σ(*F*^2^)] = 0.061	H-atom parameters constrained
*wR*(*F*^2^) = 0.133	*w* = 1/[σ^2^(*F*_o_^2^) + (0.0486*P*)^2^] where *P* = (*F*_o_^2^ + 2*F*_c_^2^)/3
*S* = 1.03	(Δ/σ)_max_ < 0.001
5559 reflections	Δρ_max_ = 0.13 e Å^−^^3^
361 parameters	Δρ_min_ = −0.18 e Å^−^^3^
Primary atom site location: structure-invariant direct methods	Extinction correction: none

### Special details

**Table:**

Geometry. Bond distances, angles *etc*. have been calculated using the rounded fractional coordinates. All su's are estimated from the variances of the (full) variance-covariance matrix. The cell e.s.d.'s are taken into account in the estimation of distances, angles and torsion angles
Refinement. Refinement on *F*^2^ for ALL reflections except those flagged by the user for potential systematic errors. Weighted *R*-factors *wR* and all goodnesses of fit *S* are based on *F*^2^, conventional *R*-factors *R* are based on *F*, with *F* set to zero for negative *F*^2^. The observed criterion of *F*^2^ > σ(*F*^2^) is used only for calculating -*R*-factor-obs *etc*. and is not relevant to the choice of reflections for refinement. *R*-factors based on *F*^2^ are statistically about twice as large as those based on *F*, and *R*-factors based on ALL data will be even larger.

### Fractional atomic coordinates and isotropic or equivalent isotropic displacement parameters (Å^2^)

**Table:**

	*x*	*y*	*z*	*U*_iso_*/*U*_eq_	
O1	0.30826 (12)	0.47676 (17)	0.36036 (9)	0.0580 (6)	
O2	0.01260 (12)	0.56344 (18)	0.12243 (10)	0.0629 (6)	
O3	0.70434 (13)	0.89046 (19)	0.54334 (10)	0.0671 (7)	
N1	0.36864 (12)	0.65460 (17)	0.32002 (9)	0.0408 (6)	
C1	0.19804 (16)	0.4947 (2)	0.18251 (11)	0.0405 (7)	
C2	0.27536 (18)	0.4117 (2)	0.17982 (13)	0.0502 (8)	
C3	0.2530 (2)	0.3274 (3)	0.12490 (15)	0.0610 (10)	
C4	0.1512 (2)	0.3219 (3)	0.07022 (15)	0.0668 (10)	
C5	0.0726 (2)	0.4008 (3)	0.07171 (14)	0.0611 (9)	
C6	0.09628 (17)	0.4865 (2)	0.12622 (12)	0.0475 (8)	
C7	0.02358 (17)	0.6316 (2)	0.18153 (15)	0.0544 (9)	
C8	−0.06802 (19)	0.6919 (3)	0.17835 (19)	0.0781 (10)	
C9	−0.0640 (2)	0.7611 (4)	0.2355 (2)	0.0939 (15)	
C10	0.0301 (2)	0.7712 (4)	0.2973 (2)	0.0964 (15)	
C11	0.1208 (2)	0.7119 (3)	0.29880 (17)	0.0729 (10)	
C12	0.12027 (16)	0.6437 (2)	0.24156 (13)	0.0489 (8)	
C13	0.22163 (15)	0.5893 (2)	0.24046 (12)	0.0412 (7)	
C14	0.30342 (16)	0.5552 (2)	0.31672 (12)	0.0410 (7)	
C15	0.30515 (15)	0.7010 (2)	0.24693 (11)	0.0401 (7)	
C16	0.45290 (15)	0.7156 (2)	0.37759 (11)	0.0397 (7)	
C17	0.52326 (16)	0.6478 (2)	0.43596 (12)	0.0466 (8)	
C18	0.60597 (16)	0.7098 (2)	0.49038 (12)	0.0501 (8)	
C19	0.61856 (16)	0.8382 (2)	0.48725 (12)	0.0474 (8)	
C20	0.54858 (18)	0.9063 (3)	0.43031 (14)	0.0578 (8)	
C21	0.46607 (17)	0.8438 (3)	0.37511 (14)	0.0572 (8)	
C22	0.7252 (2)	1.0209 (3)	0.53897 (18)	0.0848 (11)	
C23	0.35611 (16)	0.7107 (2)	0.19465 (11)	0.0416 (7)	
C24	0.46163 (17)	0.6711 (2)	0.20799 (12)	0.0447 (7)	
C25	0.52860 (17)	0.5941 (2)	0.26651 (13)	0.0498 (8)	
C26	0.63122 (19)	0.5661 (2)	0.27846 (16)	0.0589 (9)	
C27	0.6760 (2)	0.6116 (3)	0.23318 (18)	0.0672 (10)	
C28	0.6161 (2)	0.6794 (3)	0.17531 (18)	0.0676 (11)	
C29	0.50704 (19)	0.7110 (2)	0.15967 (14)	0.0535 (9)	
C30	0.4453 (2)	0.7773 (3)	0.09829 (16)	0.0650 (10)	
C31	0.3389 (2)	0.8062 (3)	0.08063 (14)	0.0609 (9)	
C32	0.2736 (3)	0.8687 (3)	0.01526 (17)	0.0853 (13)	
C33	0.1714 (3)	0.8974 (4)	−0.00129 (18)	0.0953 (15)	
C34	0.1254 (2)	0.8668 (3)	0.04666 (17)	0.0819 (11)	
C35	0.18394 (19)	0.8077 (3)	0.11000 (14)	0.0622 (9)	
C36	0.29327 (18)	0.7731 (2)	0.12966 (13)	0.0500 (8)	
H2	0.34400	0.41390	0.21640	0.0600*	
H3	0.30630	0.27400	0.12440	0.0730*	
H4	0.13580	0.26510	0.03270	0.0800*	
H5	0.00350	0.39590	0.03580	0.0730*	
H8	−0.13180	0.68530	0.13750	0.0940*	
H9	−0.12520	0.80210	0.23290	0.1130*	
H10	0.03220	0.81640	0.33660	0.1150*	
H11	0.18440	0.71840	0.33990	0.0870*	
H15	0.27310	0.78260	0.24940	0.0480*	
H17	0.51490	0.56100	0.43850	0.0560*	
H18	0.65350	0.66430	0.52940	0.0600*	
H20	0.55610	0.99340	0.42850	0.0690*	
H21	0.41910	0.88950	0.33590	0.0690*	
H22A	0.66410	1.06950	0.53510	0.1020*	
H22B	0.73910	1.03550	0.49760	0.1020*	
H22C	0.78640	1.04570	0.58120	0.1020*	
H25	0.50130	0.56190	0.29750	0.0600*	
H26	0.67230	0.51580	0.31740	0.0710*	
H27	0.74720	0.59480	0.24330	0.0810*	
H28	0.64560	0.70650	0.14450	0.0810*	
H30	0.47600	0.80320	0.06790	0.0780*	
H32	0.30300	0.88990	−0.01660	0.1020*	
H33	0.13040	0.93760	−0.04430	0.1150*	
H34	0.05430	0.88710	0.03480	0.0980*	
H35	0.15240	0.78970	0.14100	0.0750*	

### Atomic displacement parameters (Å^2^)

**Table:**

	*U*^11^	*U*^22^	*U*^33^	*U*^12^	*U*^13^	*U*^23^
O1	0.0620 (10)	0.0613 (11)	0.0500 (10)	−0.0043 (8)	0.0228 (8)	0.0149 (9)
O2	0.0431 (8)	0.0711 (12)	0.0598 (11)	0.0029 (8)	0.0070 (8)	−0.0105 (10)
O3	0.0571 (10)	0.0691 (13)	0.0525 (11)	−0.0128 (9)	0.0007 (8)	−0.0011 (10)
N1	0.0390 (9)	0.0453 (11)	0.0344 (10)	−0.0005 (8)	0.0117 (7)	0.0030 (9)
C1	0.0445 (11)	0.0412 (12)	0.0388 (12)	−0.0027 (9)	0.0203 (10)	0.0034 (10)
C2	0.0519 (12)	0.0503 (14)	0.0499 (14)	0.0033 (11)	0.0229 (11)	0.0037 (12)
C3	0.0782 (17)	0.0555 (16)	0.0585 (17)	0.0071 (13)	0.0376 (14)	0.0002 (14)
C4	0.0913 (19)	0.0581 (17)	0.0512 (16)	−0.0055 (15)	0.0302 (15)	−0.0113 (14)
C5	0.0646 (15)	0.0624 (17)	0.0487 (16)	−0.0079 (13)	0.0163 (12)	−0.0054 (14)
C6	0.0483 (12)	0.0487 (14)	0.0427 (13)	−0.0020 (10)	0.0164 (10)	0.0013 (11)
C7	0.0411 (12)	0.0551 (15)	0.0635 (17)	−0.0023 (10)	0.0186 (11)	−0.0073 (13)
C8	0.0388 (13)	0.077 (2)	0.105 (2)	0.0015 (12)	0.0170 (14)	−0.0199 (19)
C9	0.0497 (15)	0.101 (3)	0.131 (3)	0.0073 (16)	0.0379 (18)	−0.034 (2)
C10	0.0661 (18)	0.119 (3)	0.111 (3)	0.0072 (18)	0.0440 (19)	−0.046 (2)
C11	0.0538 (14)	0.093 (2)	0.0724 (19)	0.0040 (14)	0.0269 (13)	−0.0246 (18)
C12	0.0409 (11)	0.0514 (14)	0.0544 (15)	−0.0019 (10)	0.0200 (11)	−0.0039 (12)
C13	0.0374 (10)	0.0424 (13)	0.0452 (13)	−0.0009 (9)	0.0187 (9)	0.0025 (11)
C14	0.0427 (11)	0.0432 (13)	0.0393 (12)	0.0028 (9)	0.0193 (9)	0.0072 (11)
C15	0.0396 (10)	0.0417 (13)	0.0342 (11)	0.0030 (9)	0.0107 (9)	0.0030 (10)
C16	0.0373 (10)	0.0465 (13)	0.0353 (11)	0.0003 (9)	0.0151 (9)	0.0018 (10)
C17	0.0476 (12)	0.0470 (14)	0.0418 (13)	0.0044 (10)	0.0155 (10)	0.0083 (11)
C18	0.0444 (12)	0.0609 (17)	0.0370 (12)	0.0066 (11)	0.0091 (10)	0.0085 (12)
C19	0.0414 (11)	0.0572 (15)	0.0377 (13)	−0.0024 (10)	0.0109 (10)	0.0001 (12)
C20	0.0557 (13)	0.0469 (14)	0.0564 (16)	−0.0039 (11)	0.0093 (12)	0.0038 (13)
C21	0.0503 (13)	0.0522 (15)	0.0494 (15)	0.0028 (11)	0.0014 (11)	0.0087 (13)
C22	0.0686 (17)	0.068 (2)	0.087 (2)	−0.0150 (15)	0.0023 (16)	−0.0109 (18)
C23	0.0483 (11)	0.0382 (12)	0.0361 (12)	−0.0086 (9)	0.0155 (10)	0.0005 (10)
C24	0.0495 (12)	0.0405 (12)	0.0464 (13)	−0.0090 (10)	0.0223 (10)	−0.0063 (11)
C25	0.0504 (12)	0.0452 (14)	0.0574 (15)	−0.0021 (10)	0.0261 (11)	−0.0039 (12)
C26	0.0509 (13)	0.0504 (15)	0.0765 (19)	−0.0004 (11)	0.0279 (13)	−0.0082 (14)
C27	0.0555 (14)	0.0589 (17)	0.095 (2)	−0.0077 (13)	0.0391 (16)	−0.0127 (17)
C28	0.0736 (17)	0.0611 (18)	0.092 (2)	−0.0195 (14)	0.0583 (17)	−0.0172 (17)
C29	0.0673 (15)	0.0474 (14)	0.0562 (16)	−0.0163 (12)	0.0362 (13)	−0.0095 (13)
C30	0.0866 (19)	0.0606 (17)	0.0637 (18)	−0.0179 (15)	0.0470 (15)	−0.0037 (15)
C31	0.0885 (18)	0.0510 (16)	0.0431 (14)	−0.0162 (14)	0.0275 (13)	−0.0003 (13)
C32	0.115 (3)	0.084 (2)	0.0501 (18)	−0.009 (2)	0.0279 (17)	0.0168 (17)
C33	0.113 (3)	0.094 (3)	0.0505 (19)	0.000 (2)	0.0063 (18)	0.0278 (19)
C34	0.0733 (18)	0.083 (2)	0.0619 (19)	−0.0001 (16)	0.0013 (15)	0.0228 (17)
C35	0.0605 (14)	0.0600 (17)	0.0518 (15)	−0.0072 (12)	0.0096 (12)	0.0109 (14)
C36	0.0583 (13)	0.0452 (14)	0.0382 (13)	−0.0091 (11)	0.0121 (10)	0.0006 (11)

### Geometric parameters (Å, °)

**Table:**

O1—C14	1.204 (3)	C26—C27	1.400 (4)
O2—C6	1.386 (3)	C27—C28	1.338 (5)
O2—C7	1.367 (3)	C28—C29	1.437 (4)
O3—C19	1.375 (3)	C29—C30	1.382 (4)
O3—C22	1.421 (4)	C30—C31	1.387 (4)
N1—C14	1.366 (3)	C31—C32	1.429 (4)
N1—C15	1.472 (3)	C31—C36	1.437 (4)
N1—C16	1.419 (3)	C32—C33	1.336 (6)
C1—C2	1.399 (3)	C33—C34	1.415 (5)
C1—C6	1.397 (3)	C34—C35	1.364 (4)
C1—C13	1.485 (3)	C35—C36	1.432 (4)
C2—C3	1.370 (4)	C2—H2	0.9300
C3—C4	1.383 (4)	C3—H3	0.9300
C4—C5	1.377 (4)	C4—H4	0.9300
C5—C6	1.372 (4)	C5—H5	0.9300
C7—C8	1.390 (4)	C8—H8	0.9300
C7—C12	1.391 (4)	C9—H9	0.9300
C8—C9	1.367 (5)	C10—H10	0.9300
C9—C10	1.388 (5)	C11—H11	0.9300
C10—C11	1.386 (5)	C15—H15	0.9800
C11—C12	1.379 (4)	C17—H17	0.9300
C12—C13	1.518 (3)	C18—H18	0.9300
C13—C14	1.543 (3)	C20—H20	0.9300
C13—C15	1.615 (3)	C21—H21	0.9300
C15—C23	1.514 (3)	C22—H22A	0.9600
C16—C17	1.386 (3)	C22—H22B	0.9600
C16—C21	1.373 (4)	C22—H22C	0.9600
C17—C18	1.383 (3)	C25—H25	0.9300
C18—C19	1.375 (3)	C26—H26	0.9300
C19—C20	1.369 (4)	C27—H27	0.9300
C20—C21	1.392 (4)	C28—H28	0.9300
C23—C24	1.423 (3)	C30—H30	0.9300
C23—C36	1.416 (3)	C32—H32	0.9300
C24—C25	1.426 (3)	C33—H33	0.9300
C24—C29	1.442 (4)	C34—H34	0.9300
C25—C26	1.361 (4)	C35—H35	0.9300
			
O1···C17	3.255 (3)	C25···H2	2.9900
O1···C18^i^	3.419 (3)	C25···H3^viii^	3.0900
O3···C8^ii^	3.317 (4)	C26···H3^viii^	2.8500
O1···H17	2.7600	C26···H21^v^	2.8500
O1···H18^i^	2.5800	C27···H21^v^	2.7800
O2···H10^iii^	2.8900	C28···H21^v^	3.1000
O3···H8^ii^	2.4100	C29···H20^v^	2.8300
O3···H28^iv^	2.7300	C30···H20^v^	3.0500
N1···C11	3.308 (4)	C35···H26^viii^	2.9200
N1···C25	2.916 (3)	C35···H15	2.6200
N1···H11	2.8100	C36···H26^viii^	2.7500
N1···H25	2.2800	H2···C14	2.7900
C1···C36	3.575 (3)	H2···C25	2.9900
C2···C23	3.326 (3)	H3···C19^v^	2.9700
C2···C27^v^	3.569 (4)	H3···C20^v^	3.0200
C3···C19^v^	3.447 (4)	H3···C25^v^	3.0900
C3···C20^v^	3.471 (4)	H3···C26^v^	2.8500
C3···C26^v^	3.389 (4)	H5···C5^vi^	2.9500
C3···C27^v^	3.509 (4)	H8···O3^vii^	2.4100
C5···C5^vi^	3.489 (4)	H8···C22^vii^	3.0700
C8···O3^vii^	3.317 (4)	H10···O2^ix^	2.8900
C11···N1	3.308 (4)	H10···C5^ix^	2.9500
C13···C35	3.414 (4)	H10···C6^ix^	2.8400
C16···C24	3.573 (3)	H11···N1	2.8100
C16···C25	3.156 (3)	H11···C14	2.5600
C17···O1	3.255 (3)	H11···C15	3.0200
C17···C25	3.564 (3)	H15···C11	2.7900
C18···O1^i^	3.419 (3)	H15···C21	2.9000
C19···C3^viii^	3.447 (4)	H15···C35	2.6200
C20···C3^viii^	3.471 (4)	H15···H21	2.3400
C23···C2	3.326 (3)	H15···H35	2.1500
C24···C16	3.573 (3)	H17···O1	2.7600
C25···C17	3.564 (3)	H17···C14	2.9400
C25···C16	3.156 (3)	H18···O1^i^	2.5800
C25···N1	2.916 (3)	H20···C22	2.5000
C26···C36^v^	3.553 (3)	H20···H22A	2.2200
C26···C3^viii^	3.389 (4)	H20···H22B	2.3600
C27···C3^viii^	3.509 (4)	H20···C29^viii^	2.8300
C27···C2^viii^	3.569 (4)	H20···C30^viii^	3.0500
C35···C13	3.414 (4)	H21···C15	2.7200
C36···C26^viii^	3.553 (3)	H21···H15	2.3400
C36···C1	3.575 (3)	H21···C26^viii^	2.8500
C5···H5^vi^	2.9500	H21···C27^viii^	2.7800
C5···H10^iii^	2.9500	H21···C28^viii^	3.1000
C6···H10^iii^	2.8400	H22A···C20	2.7000
C7···H35	2.8100	H22A···H20	2.2200
C11···H15	2.7900	H22B···C20	2.7600
C12···H35	2.7600	H22B···H20	2.3600
C13···H35	2.8200	H25···N1	2.2800
C14···H25	2.9100	H25···C14	2.9100
C14···H11	2.5600	H25···C15	2.8600
C14···H17	2.9400	H25···C16	2.5900
C14···H2	2.7900	H25···C17	2.8800
C15···H21	2.7200	H26···C35^v^	2.9200
C15···H35	2.4900	H26···C36^v^	2.7500
C15···H25	2.8600	H28···H30	2.4300
C15···H11	3.0200	H28···O3^x^	2.7300
C16···H25	2.5900	H30···H28	2.4300
C17···H30^iv^	3.0900	H30···H32	2.4600
C17···H25	2.8800	H30···C17^x^	3.0900
C18···H30^iv^	2.8600	H30···C18^x^	2.8600
C19···H3^viii^	2.9700	H32···H30	2.4600
C20···H22A	2.7000	H35···C7	2.8100
C20···H3^viii^	3.0200	H35···C12	2.7600
C20···H22B	2.7600	H35···C13	2.8200
C21···H15	2.9000	H35···C15	2.4900
C22···H8^ii^	3.0700	H35···H15	2.1500
C22···H20	2.5000		
			
C6—O2—C7	118.7 (2)	C30—C31—C32	121.8 (3)
C19—O3—C22	117.6 (2)	C30—C31—C36	119.1 (3)
C14—N1—C15	96.30 (16)	C32—C31—C36	119.1 (3)
C14—N1—C16	133.00 (18)	C31—C32—C33	121.7 (3)
C15—N1—C16	128.98 (18)	C32—C33—C34	120.1 (3)
C2—C1—C6	116.3 (2)	C33—C34—C35	120.9 (3)
C2—C1—C13	122.8 (2)	C34—C35—C36	121.2 (3)
C6—C1—C13	120.9 (2)	C23—C36—C31	120.2 (2)
C1—C2—C3	122.0 (2)	C23—C36—C35	122.7 (2)
C2—C3—C4	120.0 (3)	C31—C36—C35	117.1 (2)
C3—C4—C5	119.6 (3)	C1—C2—H2	119.00
C4—C5—C6	120.0 (3)	C3—C2—H2	119.00
O2—C6—C1	122.1 (2)	C2—C3—H3	120.00
O2—C6—C5	115.8 (2)	C4—C3—H3	120.00
C1—C6—C5	122.1 (2)	C3—C4—H4	120.00
O2—C7—C8	116.1 (3)	C5—C4—H4	120.00
O2—C7—C12	123.1 (2)	C4—C5—H5	120.00
C8—C7—C12	120.7 (3)	C6—C5—H5	120.00
C7—C8—C9	119.8 (3)	C7—C8—H8	120.00
C8—C9—C10	121.0 (3)	C9—C8—H8	120.00
C9—C10—C11	118.1 (3)	C8—C9—H9	119.00
C10—C11—C12	122.4 (3)	C10—C9—H9	119.00
C7—C12—C11	117.9 (2)	C9—C10—H10	121.00
C7—C12—C13	119.7 (2)	C11—C10—H10	121.00
C11—C12—C13	122.4 (2)	C10—C11—H11	119.00
C1—C13—C12	112.00 (19)	C12—C11—H11	119.00
C1—C13—C14	118.68 (18)	N1—C15—H15	109.00
C1—C13—C15	117.93 (19)	C13—C15—H15	109.00
C12—C13—C14	110.92 (19)	C23—C15—H15	109.00
C12—C13—C15	110.36 (17)	C16—C17—H17	120.00
C14—C13—C15	84.07 (16)	C18—C17—H17	120.00
O1—C14—N1	131.6 (2)	C17—C18—H18	120.00
O1—C14—C13	135.1 (2)	C19—C18—H18	120.00
N1—C14—C13	93.13 (17)	C19—C20—H20	120.00
N1—C15—C13	86.41 (15)	C21—C20—H20	120.00
N1—C15—C23	119.70 (19)	C16—C21—H21	119.00
C13—C15—C23	121.11 (18)	C20—C21—H21	120.00
N1—C16—C17	121.18 (19)	O3—C22—H22A	109.00
N1—C16—C21	119.6 (2)	O3—C22—H22B	109.00
C17—C16—C21	119.3 (2)	O3—C22—H22C	109.00
C16—C17—C18	119.7 (2)	H22A—C22—H22B	109.00
C17—C18—C19	120.6 (2)	H22A—C22—H22C	110.00
O3—C19—C18	116.1 (2)	H22B—C22—H22C	109.00
O3—C19—C20	123.7 (2)	C24—C25—H25	119.00
C18—C19—C20	120.2 (2)	C26—C25—H25	119.00
C19—C20—C21	119.3 (3)	C25—C26—H26	119.00
C16—C21—C20	121.0 (3)	C27—C26—H26	119.00
C15—C23—C24	125.59 (19)	C26—C27—H27	120.00
C15—C23—C36	114.9 (2)	C28—C27—H27	120.00
C24—C23—C36	119.3 (2)	C27—C28—H28	119.00
C23—C24—C25	125.3 (2)	C29—C28—H28	119.00
C23—C24—C29	118.9 (2)	C29—C30—H30	119.00
C25—C24—C29	115.9 (2)	C31—C30—H30	119.00
C24—C25—C26	122.1 (2)	C31—C32—H32	119.00
C25—C26—C27	121.2 (3)	C33—C32—H32	119.00
C26—C27—C28	119.9 (3)	C32—C33—H33	120.00
C27—C28—C29	121.4 (3)	C34—C33—H33	120.00
C24—C29—C28	119.4 (2)	C33—C34—H34	120.00
C24—C29—C30	120.1 (3)	C35—C34—H34	120.00
C28—C29—C30	120.5 (3)	C34—C35—H35	119.00
C29—C30—C31	121.9 (3)	C36—C35—H35	119.00
			
C7—O2—C6—C5	166.4 (2)	C12—C13—C14—N1	−107.24 (19)
C6—O2—C7—C8	−170.7 (2)	C1—C13—C14—N1	121.0 (2)
C7—O2—C6—C1	−12.9 (3)	C1—C13—C15—N1	−121.6 (2)
C6—O2—C7—C12	10.6 (3)	C15—C13—C14—N1	2.31 (16)
C22—O3—C19—C18	174.8 (2)	C14—C13—C15—N1	−2.15 (15)
C22—O3—C19—C20	−4.6 (4)	C14—C13—C15—C23	120.7 (2)
C15—N1—C16—C17	−162.1 (2)	C12—C13—C15—C23	−129.2 (2)
C15—N1—C14—C13	−2.54 (18)	C15—C13—C14—O1	178.3 (3)
C14—N1—C16—C21	−144.4 (3)	C13—C15—C23—C24	−101.0 (3)
C16—N1—C15—C13	−164.0 (2)	C13—C15—C23—C36	83.6 (2)
C16—N1—C14—C13	163.0 (2)	N1—C15—C23—C24	4.0 (3)
C14—N1—C15—C13	2.43 (17)	N1—C15—C23—C36	−171.31 (19)
C16—N1—C15—C23	72.0 (3)	N1—C16—C21—C20	−179.3 (2)
C15—N1—C14—O1	−178.8 (3)	C17—C16—C21—C20	−0.2 (4)
C16—N1—C14—O1	−13.3 (4)	N1—C16—C17—C18	178.5 (2)
C14—N1—C15—C23	−121.6 (2)	C21—C16—C17—C18	−0.6 (4)
C14—N1—C16—C17	36.5 (4)	C16—C17—C18—C19	0.4 (4)
C15—N1—C16—C21	17.0 (3)	C17—C18—C19—C20	0.5 (4)
C2—C1—C13—C14	−34.3 (3)	C17—C18—C19—O3	−178.9 (2)
C6—C1—C13—C15	−113.7 (2)	O3—C19—C20—C21	178.1 (2)
C6—C1—C13—C14	147.3 (2)	C18—C19—C20—C21	−1.3 (4)
C13—C1—C6—C5	179.4 (2)	C19—C20—C21—C16	1.1 (4)
C2—C1—C13—C12	−165.6 (2)	C15—C23—C24—C25	13.0 (3)
C2—C1—C13—C15	64.7 (3)	C15—C23—C24—C29	−166.6 (2)
C6—C1—C13—C12	16.0 (3)	C36—C23—C24—C25	−171.8 (2)
C2—C1—C6—C5	0.9 (3)	C36—C23—C24—C29	8.6 (3)
C13—C1—C6—O2	−1.4 (3)	C15—C23—C36—C31	170.1 (2)
C2—C1—C6—O2	−179.9 (2)	C15—C23—C36—C35	−9.0 (3)
C6—C1—C2—C3	0.4 (4)	C24—C23—C36—C31	−5.6 (3)
C13—C1—C2—C3	−178.1 (2)	C24—C23—C36—C35	175.4 (2)
C1—C2—C3—C4	−0.5 (4)	C23—C24—C25—C26	−175.9 (2)
C2—C3—C4—C5	−0.5 (5)	C29—C24—C25—C26	3.7 (3)
C3—C4—C5—C6	1.7 (4)	C23—C24—C29—C28	175.5 (2)
C4—C5—C6—C1	−2.0 (4)	C23—C24—C29—C30	−5.5 (3)
C4—C5—C6—O2	178.8 (2)	C25—C24—C29—C28	−4.1 (3)
O2—C7—C8—C9	179.4 (3)	C25—C24—C29—C30	174.8 (2)
O2—C7—C12—C11	−178.2 (2)	C24—C25—C26—C27	−0.3 (4)
O2—C7—C12—C13	5.5 (3)	C25—C26—C27—C28	−2.9 (4)
C12—C7—C8—C9	−1.8 (4)	C26—C27—C28—C29	2.3 (5)
C8—C7—C12—C13	−173.2 (2)	C27—C28—C29—C24	1.3 (4)
C8—C7—C12—C11	3.2 (4)	C27—C28—C29—C30	−177.7 (3)
C7—C8—C9—C10	−0.7 (5)	C24—C29—C30—C31	−0.8 (4)
C8—C9—C10—C11	1.7 (6)	C28—C29—C30—C31	178.2 (3)
C9—C10—C11—C12	−0.2 (5)	C29—C30—C31—C32	−176.5 (3)
C10—C11—C12—C7	−2.2 (4)	C29—C30—C31—C36	3.9 (4)
C10—C11—C12—C13	174.1 (3)	C30—C31—C32—C33	−179.4 (3)
C11—C12—C13—C15	−60.6 (3)	C36—C31—C32—C33	0.2 (5)
C7—C12—C13—C14	−153.1 (2)	C30—C31—C36—C23	−0.7 (4)
C7—C12—C13—C1	−18.0 (3)	C30—C31—C36—C35	178.4 (3)
C11—C12—C13—C14	30.8 (3)	C32—C31—C36—C23	179.8 (2)
C7—C12—C13—C15	115.6 (2)	C32—C31—C36—C35	−1.2 (4)
C11—C12—C13—C1	165.9 (2)	C31—C32—C33—C34	0.5 (5)
C12—C13—C15—N1	107.99 (19)	C32—C33—C34—C35	−0.2 (5)
C1—C13—C15—C23	1.3 (3)	C33—C34—C35—C36	−0.9 (5)
C1—C13—C14—O1	−63.0 (4)	C34—C35—C36—C23	−179.4 (3)
C12—C13—C14—O1	68.8 (3)	C34—C35—C36—C31	1.5 (4)

Symmetry codes: (i) −*x*+1, −*y*+1, −*z*+1; (ii) *x*+1, −*y*+3/2, *z*+1/2; (iii) −*x*, *y*−1/2, −*z*+1/2; (iv) *x*, −*y*+3/2, *z*+1/2; (v) −*x*+1, *y*−1/2, −*z*+1/2; (vi) −*x*, −*y*+1, −*z*; (vii) *x*−1, −*y*+3/2, *z*−1/2; (viii) −*x*+1, *y*+1/2, −*z*+1/2; (ix) −*x*, *y*+1/2, −*z*+1/2; (x) *x*, −*y*+3/2, *z*−1/2.

### Hydrogen-bond geometry (Å, °)

**Table:**

*D*—H···*A*	*D*—H	H···*A*	*D*···*A*	*D*—H···*A*
C8—H8···O3^vii^	0.93	2.41	3.317 (4)	164
C18—H18···O1^i^	0.93	2.58	3.419 (3)	150
C25—H25···N1	0.93	2.28	2.916 (3)	125

Symmetry codes: (vii) *x*−1, −*y*+3/2, *z*−1/2; (i) −*x*+1, −*y*+1, −*z*+1.
